# Data Preprocessing Techniques for AI and Machine Learning Readiness: Scoping Review of Wearable Sensor Data in Cancer Care

**DOI:** 10.2196/59587

**Published:** 2024-09-27

**Authors:** Bengie L Ortiz, Vibhuti Gupta, Rajnish Kumar, Aditya Jalin, Xiao Cao, Charles Ziegenbein, Ashutosh Singhal, Muneesh Tewari, Sung Won Choi

**Affiliations:** 1 Department of Pediatrics, Hematology and Oncology Division, Michigan Medicine, University of Michigan Health System Ann Arbor, MI United States; 2 School of Applied Computational Sciences, Meharry Medical College Nashville, TN United States; 3 Autonomous Systems Research Department, Peraton Labs Basking Ridge, NJ United States; 4 Department of Biomedical Engineering, College of Engineering, University of Michigan Ann Arbor, MI United States; 5 Rogel Comprehensive Cancer Center, University of Michigan Ann Arbor, MI United States; 6 VA Ann Arbor Healthcare System Ann Arbor, MI United States; 7 Center for Computational Medicine and Bioinformatics, University of Michigan Ann Arbor, MI United States; 8 Department of Internal Medicine, University of Michigan Ann Arbor, MI United States

**Keywords:** machine learning, artificial intelligence, preprocessing, wearables, mobile phone, cancer care

## Abstract

**Background:**

Wearable sensors are increasingly being explored in health care, including in cancer care, for their potential in continuously monitoring patients. Despite their growing adoption, significant challenges remain in the quality and consistency of data collected from wearable sensors. Moreover, preprocessing pipelines to clean, transform, normalize, and standardize raw data have not yet been fully optimized.

**Objective:**

This study aims to conduct a scoping review of preprocessing techniques used on raw wearable sensor data in cancer care, specifically focusing on methods implemented to ensure their readiness for artificial intelligence and machine learning (AI/ML) applications. We sought to understand the current landscape of approaches for handling issues, such as noise, missing values, normalization or standardization, and transformation, as well as techniques for extracting meaningful features from raw sensor outputs and converting them into usable formats for subsequent AI/ML analysis.

**Methods:**

We systematically searched IEEE Xplore, PubMed, Embase, and Scopus to identify potentially relevant studies for this review. The eligibility criteria included (1) mobile health and wearable sensor studies in cancer, (2) written and published in English, (3) published between January 2018 and December 2023, (4) full text available rather than abstracts, and (5) original studies published in peer-reviewed journals or conferences.

**Results:**

The initial search yielded 2147 articles, of which 20 (0.93%) met the inclusion criteria. Three major categories of preprocessing techniques were identified: data transformation (used in 12/20, 60% of selected studies), data normalization and standardization (used in 8/20, 40% of the selected studies), and data cleaning (used in 8/20, 40% of the selected studies). Transformation methods aimed to convert raw data into more informative formats for analysis, such as by segmenting sensor streams or extracting statistical features. Normalization and standardization techniques usually normalize the range of features to improve comparability and model convergence. Cleaning methods focused on enhancing data reliability by handling artifacts like missing values, outliers, and inconsistencies.

**Conclusions:**

While wearable sensors are gaining traction in cancer care, realizing their full potential hinges on the ability to reliably translate raw outputs into high-quality data suitable for AI/ML applications. This review found that researchers are using various preprocessing techniques to address this challenge, but there remains a lack of standardized best practices. Our findings suggest a pressing need to develop and adopt uniform data quality and preprocessing workflows of wearable sensor data that can support the breadth of cancer research and varied patient populations. Given the diverse preprocessing techniques identified in the literature, there is an urgency for a framework that can guide researchers and clinicians in preparing wearable sensor data for AI/ML applications. For the scoping review as well as our research, we propose a general framework for preprocessing wearable sensor data, designed to be adaptable across different disease settings, moving beyond cancer care.

## Introduction

### Background

According to the US Food and Drug Administration, digital health is categorized as *mobile health* (mHealth), health information technology, wearable devices, telehealth, personalized medicine, and telemedicine [1]. Digital health has revolutionized health care by offering the potential for continuous and noninvasive monitoring of human physiological parameters, such as heart rate, sleep, and activity levels, to facilitate the early detection and prevention of life-threatening diseases [2]. Digital health consists of collecting, analyzing, storing, and sharing health care data by harnessing the power of technology, including smartphone apps, wearable sensors, telemedicine, the Internet of Medical Things, etc [3]. Due to the widespread use of mHealth technologies and routine use of wearable sensors (eg, smartwatches), the person-generated health data have become promising data sources for biomedical research [4].

Indeed, the integration of wearable sensors into cancer care has opened new pathways for remote monitoring, enabling health care providers to gather a wealth of real-time data from patients [5-7]. These wearables capture an array of physiological parameters, including skin temperature [8], offering insights into the patient’s response to cancer treatment, quality of life, and overall well-being [9]. These continuous streams of data have the potential to transform cancer care by providing an improved understanding of patient conditions outside of the hospital setting, potentially improving clinical outcomes. Nevertheless, transforming raw data into meaningful analysis and insights presents numerous challenges, making standardized workflows for data preprocessing essential.

Data preprocessing involves a series of steps designed to clean and refine data to ensure its reliability and suitability for analysis using artificial intelligence and machine learning (AI/ML) techniques. The preprocessing steps help transform raw sensor data, which can be noisy and inconsistent, into a clean, structured format suitable for AI/ML models to process [10-12]. Without standardization in these procedures, there is a risk that subsequent data analysis might be based on flawed information, leading to uninterpretable data, a lack of generalizability, and erroneous conclusions. Typical preprocessing steps to make sensor data AI/ML ready include data cleaning (eg, noise reduction, outlier detection, and handling missing data) [13,14], data integration (eg, combining data sources and aligning time stamps), data transformation (eg, windowing and normalization) [15], dimensionality reduction (eg, feature selection), and data labeling (eg, annotating).

AI/ML’s scope has become an amazing supportive tool for digital health [16,17] since its potential evolution to exploit meaningful relationships in biomedical data sets that can be used for diagnosis, prediction, and treatments [18-21]. AI/ML techniques have become popular in biometrics extraction mobile apps smart systems, such as eye disease detection [22-24], atrial fibrillation [25], heart rate monitoring [26], etc. In addition, a summary of the actual cancer statistics and its future directions is provided in the study by Moher et al [27].

Within the integration of electronic health record technology [26] in digital medicine, wearable monitoring devices have earned an important and crucial role for all people in the biomedical area (eg, patients, medical staff, and biomedical researchers). Oncology divisions have ultimately contemplated the importance of incorporating mHealth monitoring while conducting clinical cancer trials [1]. Moreover, multiple types of cancer disease detection using AI/ML techniques are a crucial factor considering its alarming impact rates on the population [27]. The mHealth integration on cancer applications for the development of AI/ML solutions has become popular in recent years [28]. However, the importance of data quality has not been highlighted while considering the design and development of prediction models. Building high-quality data is a critical step while applying AI/ML algorithms in mHealth and wearable studies; however, the emphasis on enriching the data quality is very limited in these studies, especially in oncology. Misclassifications, misdiagnoses, and wrong predictions can be avoided, and the whole mHealth system feasibility can be improved by enriching the data quality.

### Goals of Our Review

This study aims to explore the use of wearable sensors for continuous monitoring of key physiological parameters in cancer care. We systematically reviewed the literature by identifying and assessing preprocessing workflows that are essential for transforming raw, noisy, and often inconsistent wearable sensor data into reliable and structured formats suitable for subsequent AI/ML modeling. By examining the current landscape of these practices, our research aims to improve wearable sensor data quality, specifically for cancer care, ensuring that downstream data analyses and interpretations are rigorous and reproducible. Given the diverse preprocessing techniques identified in the literature, there is an urgency for a framework that can guide researchers and clinicians in preparing wearable sensor data for AI/ML applications. This paper proposes a framework designed to be adaptable across different continuous monitoring applications.

## Methods

### Search Strategy

We conducted a scoping review of articles written in English using the following literature databases: IEEE Xplore, PubMed, Embase, and Scopus, while following the PRISMA-ScR (Preferred Reporting Items for Systematic reviews and Meta-Analyses extension for Scoping Reviews) guidelines [29]. We have used Covidence (Veritas Health Innovation Ltd) [30] for identification and screening stages. The search was performed on December 31, 2023, using the search queries shown in Multimedia Appendix 1. We selected full peer-reviewed publications from the last 5 years (from January 2018 to December 2023), focusing on preprocessing techniques used on wearable sensor data to ensure their readiness for AI/ML applications for different cancer populations. Searches were developed using 3 key concepts: wearable devices, AI/ML, and cancer. Controlled vocabulary and keywords were selected for the specific databases.

**Figure 1:**
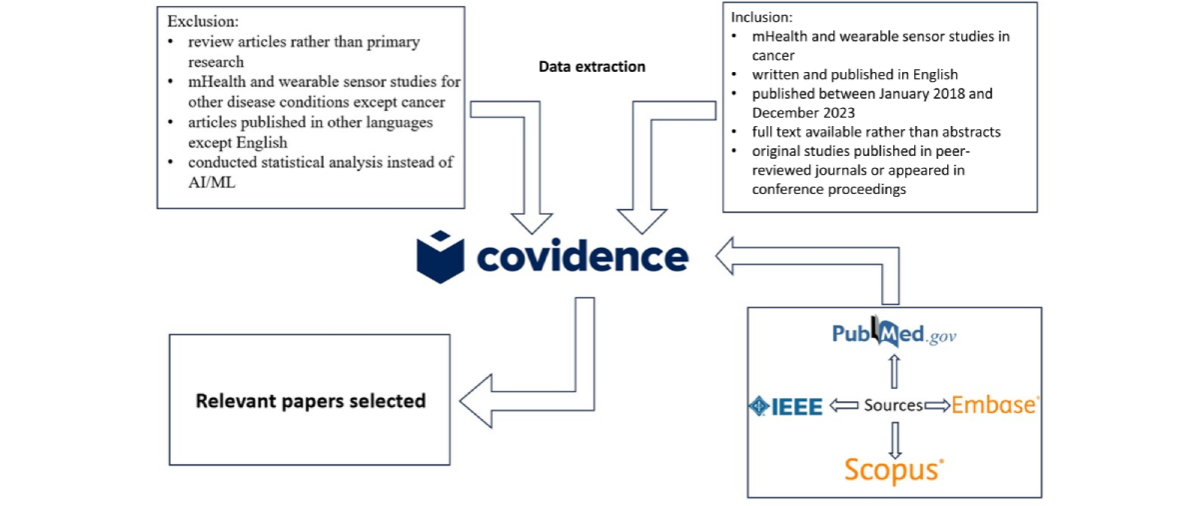
Illustration of the study selection process. AI/ML: artificial intelligence and machine learning; mHealth: mobile health.

Figure 1 shows an illustration of the study selection process for this paper. The identified studies meeting the inclusion criteria were subsequently organized based on the major themes identified.

### Inclusion Criteria

**Figure 2:**
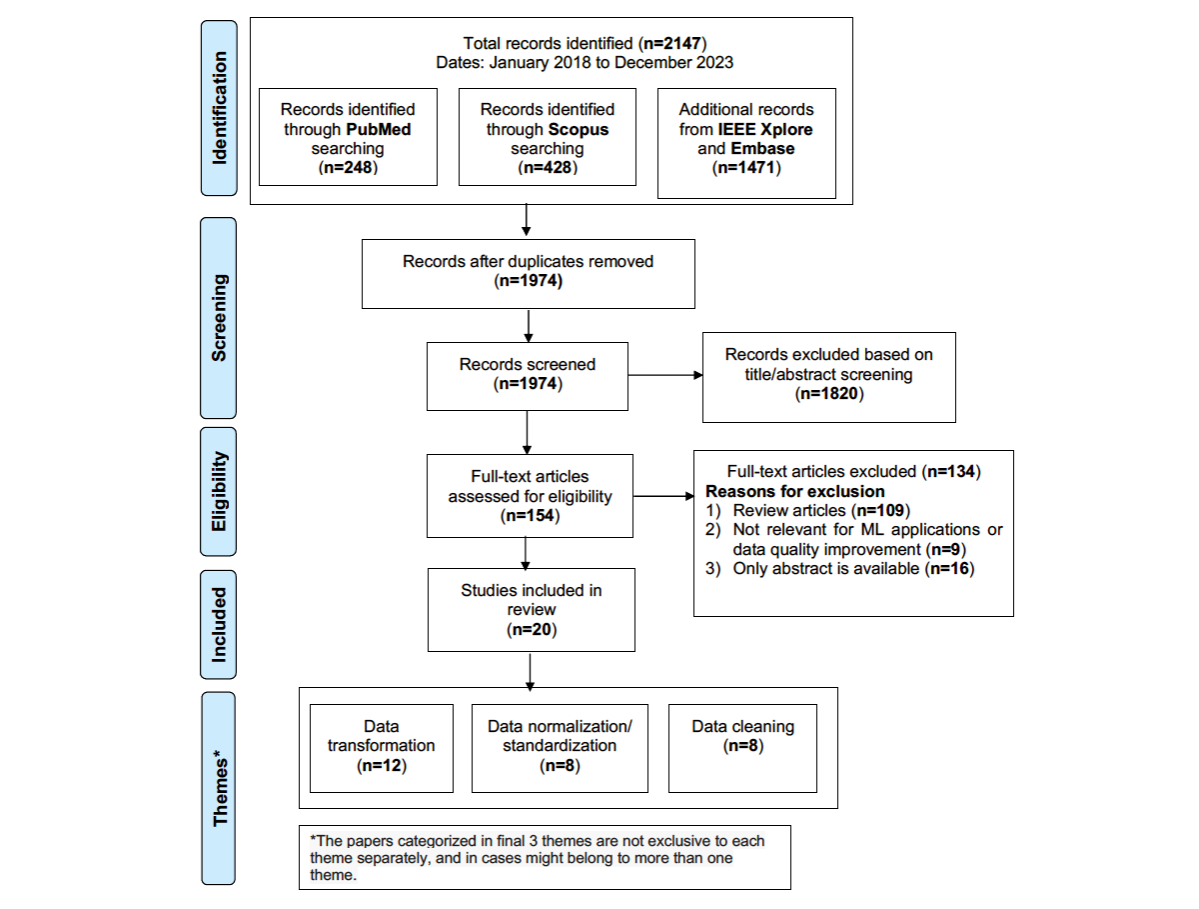
PRISMA-ScR (Preferred Reporting Items for Systematic reviews and Meta-Analyses extension for Scoping Reviews) diagram for a scoping review of biomedical scientific literature. ML: machine learning.

Our results with the search query presented in Multimedia Appendix 1 were first imported into Covidence for screening. The title and abstracts of the resulting studies were screened to identify the studies related to preprocessing techniques for wearable sensor data in cancer. After identifying the eligible studies, additional inclusion exclusion criteria were applied to retrieve the primary studies of our review (Figure 2 in the *Results* section). Studies were eligible if they fulfilled the following inclusion criteria in our review: (1) mHealth and wearable sensor studies in cancer, (2) written and published in English, (3) published between January 2018 and December 2023, (4) full text available rather than abstracts, and (5) original studies published in peer-reviewed journals or appeared in conference proceedings. PRISMA-ScR checklist is provided in Multimedia Appendix 2.

### Exclusion Criteria

Studies were not eligible if they fulfilled the following exclusion criteria in our review: (1) review articles rather than primary research, (2) mHealth and wearable sensor studies for other disease conditions except cancer, (3) articles published in other languages except English, and (4) conducted statistical analysis instead of AI/ML.

### Data Extraction and Evaluation

The data were extracted from all studies meeting our inclusion criteria for the review and organized into tables containing each study’s information (eg, authors’ name, title, and year of publication), wearable sensor data collected in cancer studies (eg, activity data, physiological parameters, including steps, sleep, heart rate, blood oxygen saturation, and temperature), preprocessing techniques (eg, time segmentation, data filtering, data transformation, and imputation), wearable devices (eg, Fitbit [Google LLC], Empatica [Empatica Inc, and Actigraphy), type of AI/ML methods applied (eg, neural networks, decision trees, K-Nearest Neighbors, Supporting Vector Machine, and regressors), sample size (eg, number of participants; Table 1). The data for all selected studies were extracted independently by 3 authors (BLO, VG, and SWC) by mutual agreement, and discrepancies were resolved by discussion with other coauthors (RK, AJ, XC, and CZ). The outcomes from the data extraction part were finally evaluated independently by each author.

**Table 1:** Summary of eligible studies.

Reference	Cancer type	Sample size, N	Wearable sensor	Physiological parameter	Preprocessing procedure	Preprocessing category	AI/ML^a^ techniques
Liu et al [30], 2023	Terminal cancer	40	Garmin VivoSmart 4	Steps, HR^b^, sleep status, and blood oxygen saturation (measured during sleep time)	Missing data imputation	Data cleaning	LR^c^, SVM^d^, DT^e^, RF^f^, KNN^g^, AdaBoost^h^, and XGBoost^i^
Zhao et al [31], 2022	Breast cancer	4	Fuschia Band prototype	Accelerometer and gyroscope readings	Peak detection and fast Fourier transform	Data transformation	KNN
Moscato et al [32], 2022	Multiple types of cancer	21	Empatica E4 wristband	Photoplethysmography signals, skin temperature, accelerometer readings, and electrodermal activity	Different- order Butterworth filtering with different cutoff frequencies and data normalization	Data cleaning and normalization and standardization	SVM, RF, MLP^j^, log, and AdaBoost
Yang et al [33], 2021	Terminal cancer	60	Actigraphy device XB40ACT	Activity level, angle, and spin	Zero padding and shortening the time series	Data transformation	LSTM^k^
Huang et al [34], 2023	Terminal cancer	78	Actigraphy device XB40ACT	Activity level, angle, and spin	Time Segmentation and zero padding	Data transformation	LSTM, bidirectional-LSTM, transformer, and GRU^l^
Cos et al [35], 2021	Pancreatic cancer	28	Fitbit inspire HR	Step count, HR, and sleep time–series data	One-hot encoding standardization and dimensionality reduction	Data transformation	RF, GBT^m^, KNN, SVM with linear kernel, and LR with L1 penalty
Davoudi et al [36], 2021	Multiple types of cancer	27	ActiGraph GT3X	Accelerometer Readings and oxygen consumption	Bias reduction, data localization, and vector magnitude calculation	Data cleaning and transformation	RF, GBT, KNN, SVM with linear kernel, and LR with L1 penalty
Liu et al [37], 2020	Multiple types of cancer	3	Fitbit Alta	HR data and activity data	Missing data imputation and data standardization	Data cleaning and normalization and standardization	Hidden Markov models
Tedesco et al [38], 2021	Multiple types of cancer	2291	ActiGraph GT3X+	Steps taken, time in light, sedentary, moderate, vigorous activities, energy expenditure, etc.	Data standardization and missing data imputation	Data cleaning and normalization and standardization	AdaBoost
Dong et al [39], 2021	Pancreatic cancer	10	ActiGraph devices	Accelerometer, light, and inclinometer	Time window segmentation	Data transformation	GRL^n^
Patel et al [40], 2023	Multiple types of cancer	50	Actiwatch	Rest-activity, sleep, and routine clinical variables	Missing data imputation with averaging technique	Data cleaning	Penalized (regularized) regression models
Asghari [41], 2021	Colorectal cancer	400	IoMT^o^ smart devices	Vital signs that were sensed through biomedical sensors	Cleaning inconsistencies and noise and Dimensionality reduction	Data cleaning and transformation	J48, SMO^p^, MLP, and NB^q^ methods
Rossi et al [42], 2021	Multiple types of cancer	52	PGHD^r^ (VivoFit)	Daily steps	Temporal segmentations	Data transformation	LR
Vets et al [43], 2023	Breast cancer	10	ActiGraph wGT3X-BT	Accelerometer readings	Counts threshold and data normalization	Data transformation and normalization and standardization	Pretrained MLM^s^
Feng et al [44], 2023	Prostate cancer	47	Google health, Fitbit, or Apple health	Step counts	Time window segmentation	Data transformation	LR
van den Eijnden et al [45], 2023	Multiple types of cancer	125	Elan sensor (wristband)	Activity features, activity counts, acceleration data, as well photoplethysmography signal	Features calculation, data dimensionality reduction and numerical to categorical data transformation, and standardization	Data transformation and normalization and standardization	LR, KNN, DT, RF, support vector regression, and XGBoost
S et al [46], 2020	Breast cancer	201	Cyrcadia breast monitor	Temperature readings	Removing outliers and missing data, duplicates removal, and data normalization	Data cleaning and normalization and standardization	DT, SVM, RF, and back propagation NN^t^
Barber et al [47], 2022	Gynecologic cancer	34	Fitbit Alta HR	Steps, HR, and intensity of physical activity	Data standardization and normalization	Data normalization and standardization	LR, RF, GBT, and XGBoost
Jacobsen et al [48], 2023	Blood cancer	79	Wearable-based RPM^u^	Time-series data recorded from biosensors	Dimensionality reduction	Data transformation	NN
Li et al [49], 2023	Multiple types of cancer	201	IMU^v^ sensor nodes, and Heal Force PC-60NW	HR and inertial measurements	Interval scaling method and z score standardization	Data normalization and standardization	MMDF^w^, XGBoost, LGBM^x^, RF, AdaBoost, and GBT

^a^AI/ML: artificial intelligence and machine learning.

^b^HR: heart rate.

^c^LR: logistic regression.

^d^SVM: support vector machine.

^e^DT: decision tree.

^f^RF: random forest.

^g^KNN: k-nearest neighbors.

^h^AdaBoost: adaptive boosting trees.

^i^XGBoost: extreme gradient boosting trees.

^j^MLP: multilayer perceptron.

^k^LSTM: long short-term memory.

^l^GRU: gated recurrent unit.

^m^GBT: gradient boosted trees.

^n^GRL: graph representation learning.

^o^IoMT: Internet of Medical Things.

^p^SMO: sequential minimal optimization.

^q^NB: naïve Bayes.

^r^PGHD: patient-generated health data.

^s^MLM: machine learning model.

^t^NN: neural network.

^u^RPM: remote patient monitoring.

^v^IMU: inertial measurement unit.

^w^MMDF: multimodel decision fusion.

^x^LGBM: light gradient boosting machine.

## Results

### Overview

**Figure 3:**
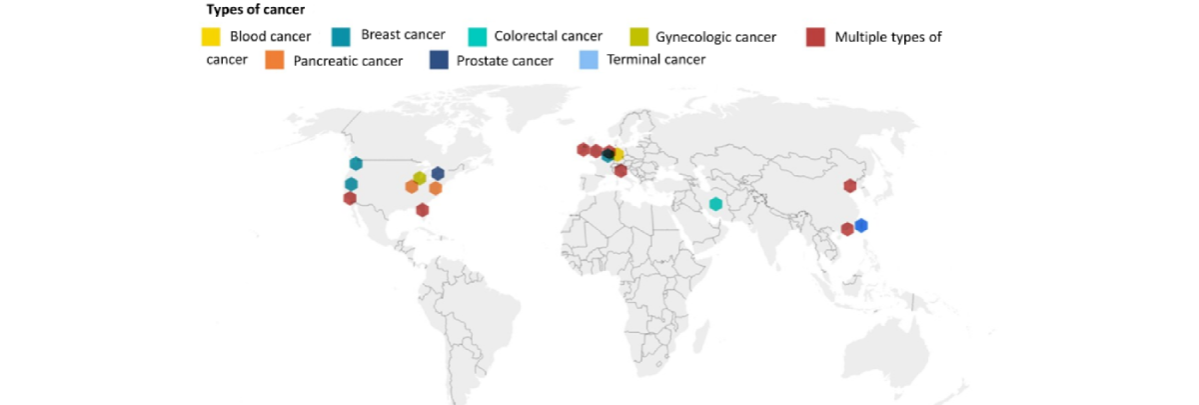
Relevant references by geographical location.

We identified 2147 studies in the initial extraction phase (n=248, 11.55% from PubMed; n=428, 19.93% from Scopus; n=996, 46.39% from IEEE Xplore; and n=475, 22.12% for Embase, including Embase, Embase Classic, MEDLINE, and PubMed-not-MEDLINE). A total of 173 (8.06%) duplicate articles were removed to produce 1974 (91.94%) for title and abstract screening. We conducted a thorough screening of titles and abstracts, which resulted in the exclusion of 1820 (92.2%) articles that did not meet the inclusion criteria. Following this screening, we identified 154 (7.8%) articles for which we performed a full-text review to assess their eligibility for inclusion in our study in more detail. In the final screening, 20 (13%) of these 154 articles met our inclusion criteria and were considered for this scoping review, as shown in Figure 2. The workflow diagram for the systematic identification of scientific literature is shown in Figure 2. The geographical distribution of these studies is mapped in Figure 3, highlighting most research from the United States. These constituted 35% (7/20) of the selected publications. Terminal cancer research was reported from Taiwan.

In terms of publication years, our analysis revealed an uptick in the frequency of papers related to mHealth and wearables in cancer. Our review coincides with the emergence of the COVID-19 pandemic, during which there was a surge in research interest within the biomedical sciences, particularly related to the use of wearable technology in remote monitoring of patients with cancer. The distribution of publications during this period suggested that in the years 2020 to 2022 combined, approximately one-quarter of the selected studies were published, accounting for 25% (5/20) of our data set. The majority were distributed between the years 2021 to 2023, which collectively contributed to 75% (15/20) of the data quality improvement strategies for wearable data preprocessing in cancer care settings. In fact, 40% (8/20) of all selected studies were published in 2023 alone, marking a substantial rise and interest in this research domain.

Our findings reported the use of wearable technology across a diverse range of cancer types. Predominantly, the category encompassing “multiple types of cancer” accounted for 40% (8/20) of the studies in this area. The remainder of the research was distributed among specific types of cancer, with each category’s contribution detailed as follows: breast cancer (3/20, 15%), terminal cancer (3/20, 15%), pancreatic cancer (2/20, 10%), blood cancer (1/20, 5%), colorectal cancer (1/20, 5%), prostate cancer (1/20, 5%), and gynecologic cancer (1/20, 5%). In addition, the recent literature indicated a trend toward increased adoption of wearable technology for cancer surveillance, signifying a growing recognition of the potential benefits that wearables may offer in continuous patient monitoring across heterogeneous cancer types.

The initial database search yielded 2147 studies, of which 20 (0.93%) met the inclusion criteria after screening and full-text review (Figure 2). The included studies applied preprocessing techniques to wearable sensor data from a range of cancer populations, including breast, colorectal, gynecologic, and blood cancers, as well as multiple other types of cancer. The most commonly used wearable devices were actigraphy sensors and consumer-grade fitness trackers, which provided data on physical activity, sleep, heart rate, and other physiological parameters.

Various preprocessing approaches are used in each of the identified themes. The most common data transformation approaches included fast Fourier transform [31], time-series segmentation [33,34,39], and statistical feature calculation [30,35,45]. However, for the data normalization techniques, *z* score standardization and min-max normalization were the most frequently reported scaling methods [32,37,43,46,49] and for the data cleaning, imputation [30,37,40], outlier removal [36,46], and artifact filtering [32,41] approaches were used. Notably, 25% (5/20) of the studies combined multiple preprocessing techniques from different categories, suggesting that a comprehensive approach to data preparation may be beneficial [32,36,38,45,46]. However, there was significant heterogeneity in the specific techniques used and their implementations across studies, highlighting a lack of standardized preprocessing pipelines for wearable sensor data in cancer care.

The preprocessing techniques were applied to support a range of AI/ML applications, including treatment response prediction [35,42], symptom monitoring [44,47], and survival analysis [33,34]. The most common ML algorithms were random forests, support vector machines, and deep learning models, such as long short-term memory networks. However, few studies directly compared the impact of different preprocessing approaches on model performance, making it difficult to draw conclusions about optimal techniques.

### Major Themes Identified

Three major themes were identified, as outlined in Table 1: (1) data normalization and standardization (8/20, 40% of papers), (2) data transformation (12/20, 60% of papers), and (3) data cleaning (8/20, 40% of papers). These were subcategorized based on the preprocessing techniques. Data transformation comprises studies related to dimensionality reduction, data feature calculation, variable transformation, or domain transformation. Data normalization and standardization included data standardization or data normalization. The data cleaning category included data filtering, outliers’ removal, imputation techniques, missing data, and duplicate removal. Multiple selected work categories were required to combine preprocessing tasks encompassing the previous 3 mentioned categories while addressing data quality issues [30-49], which are presented in Tables 1 and 2.

**Table 2:** A summary of relevant preprocessing elements on selected published works.

Reference	Time resolution	Exclusion criteria	Missing data imputation technique	Features extracted	Outcomes
Liu et al [30], 2023	Each day was a data point	Days with no wearable device data uploaded	Linear interpolation	A combination of basic demographic data, clinical assessment data, and wearable device data	Death event prediction
Zhao et al [31], 2022	Data were sent at a rate of 4 times per s	Determine whether an exercise is completed correctly or incorrectly	Not applicable	Statistical gyroscopic-based features obtained from all 3 axes (x, y, and z)	Rehabilitation
Moscato et al [32], 2022	A 2-min time window before the beginning of each session was created	Feature pairing was tested by Pearson correlation coefficient >0.9	Linear interpolation	12 features from the HRV^a^ analysis, 5 features from the photoplethysmography morphological analysis, 17 features from the electrodermal activity, 3 features from the temperature, and 2 features from the activity index	Pain assessment
Yang et al [33], 2021	An average value of 20 timesteps within total time shortened to <500 timesteps	Time series >500 timesteps	Zero paddings until the maximum length of the time series was reached	Physical activity, angle, and spin	Survival prediction
Huang et al [34], 2023	A mean of 20 timesteps was chosen as the average value for 3 time frames (12, 24, and 48 h)	Properly designed patients’ admission criteria	Zero padding was used to reach the maximum length of the time series	Physical activity, angle, and spin and the clinical data from patients were also considered	Survival prediction
Cos et al [35], 2021	Biobehavioral rhythmic features were computed for the entire tested period, and statistical and semantic features were generated daily	Biobehavioral rhythmic features were excluded due to the dimensions	Data-level and feature-level	First- and second-order statistical features from the daily step count, HR^b^, and sleep time–series data	Pancreatectomy treatment outcomes from patients activity
Davoudi et al [36], 2021	Extracted relevant features from a 16-s window; data were eventually smoothed with a 30-s running average window	Data length <4 min	Not applicable	Time and frequency domain features	Physical activity recognition and energy expenditure estimation
Liu et al [37], 2020	Disaggregating the 15-min step count data and simulating the 1-min step count time series	Nonwear days were identified and removed before the analysis	Thresholding	Statistics from HR metrics and activity levels	Algorithm validation
Tedesco et al [38], 2021	Not provided	Wear time per day was <600 min	Feature mean	Statistical features from (1) demographics, (2) self-report health and lifestyle, (3) wearable data, and (4) laboratory tests	Cancer- specific mortality prediction
Dong et al [39], 2021	1-min epoch to aggregate and synchronize the raw actigraphy data	9.5 h window size for accelerometer data to fit models	Not applicable	Time and frequency domain features from actigraphy and laboratory tests	Salivary cortisol levels on in patients with pancreatic cancer
Patel et al [40], 2023	Numerical continuous variables involving sleep-wake times were entered in the 24 h format	Data were excluded from the 1-h period before and after going to bed	Average values	Sleep-based features and sleep-wake transitional-related features	Exploratory machine learning study
Asghari [41], 2021	Not provided	Data inconsistencies removal	Not applicable	Demographics, clinical features, and wearable data	Diagnostic prediction on CRC^c^ older adults
Rossi et al [42], 2021	Three distinct types of temporal segments for weekly observations	Periods before admission	Majority class	Activity or steps related features and clinical data	Postsurgery complications
Vets et al [43], 2023	Acceleration data’s sampling rate was 30 Hz	Unknown data were discarded from further analysis	Spline interpolation	Statistical parameters from accelerometer readings	Rehabilitation study
Feng et al [44], 2023	A window of 48 h following step count decline	A decline of 1000 steps or more as a binary predictor among participants	Thresholding	Step counts calculated on different time windows	Physical activity monitoring on active treatment
van den Eijnden et al [45], 2023	The data were stored at 1-s intervals	Early stopping algorithm	Not applicable	For health dot sensor: RR^d^, activity level (actlevel); for Elan wristband: statistical parameters from HR, and frequency domain features	Recovery scores
S et al [46], 2020	Temperature profiles had values from 16 sensors gathered for 1 d at every 5-min interval	Out-of-range temperature data discrimination	Not applicable	Linear and nonlinear features from the time-series temperature data	Introductory paper
Barber et al [47], 2022	Each day was considered an observation	Discrimination of days was applied to unscheduled contacts	Not applicable	Fatigue, physical function, anxiety, mean daily HR, daily steps, sleep, and time-related features	Feasibility and events prediction
Jacobsen et al [48], 2023	Raw signals were acquired with a frequency of >30 Hz; calculated parameters were stored with a rate of 1 Hz	Data points reduction due to interruptions	Not applicable	Noninvasive monitoring of vital signs and physical activity; SCC^e^ events	Clinical complications during treatment
Li et al [49], 2023	Sampling frequency was 200 Hz for IMU^f^; the HR was stored at a sampling frequency was 1 Hz	Feature selection for redundancy removal	Majority class	HR metrics, physical activity parameters, Blood Mass Index, and blood oxygen statistical values	Physical fitness assessment

^a^HRV: heart rate variability.

^b^HR: heart rate.

^c^CRC: colorectal cancer.

^d^RR: respiratory rate.

^e^SCC: serious clinical complications.

^f^IMU: inertial measurement unit.

### Data Transformation

Zhao et al [31] reported a proof-of-concept for postoperative rehabilitation in a small cohort of 4 patients with breast cancer, using a prototype that used peak detection and Fourier transform by switching time domain points of the 3D axis to a predetermined frequency. Yang et al [33] hypothesized that wristband actigraphy monitoring devices could predict in-hospital death of end-stage multiple types of patients with cancer during the hospitalization period admissions. To avoid variations in each patient’s data length, zero padding was used until the maximum length of the time series was reached [33]. Scoring systems, such as the Palliative Prognostic Index and Palliative Performance Scale, were considered for fitting machine learning models (MLMs) [33]. Huang et al [34] reported a comparison study between the results of wearable-based activity monitoring with traditional prognostic tools for patients with end-stage cancer. In total 3 different time frames were segmented for preprocessing [34]. A mean of 20 timesteps was selected as the average value for each of the 3 different time frames (48, 24, and 12 h) [34]. Zero padding was used in the study by Huang et al [34], making it applicable to data transformation. Cos et al [35] used a wearable device to predict treatment outcomes in patients with pancreatic cancer, standardizing data before using ML methods.

Dong et al [39] proposed a general predictive modeling process that used actigraphy data to predict underlying salivary cortisol levels using graph representation learning. The raw sensor data were preprocessed using time window segmentation to reduce noise in the data [39]. Rossi et al [42] focused on predicting postdischarge oncologic surgical complications and their impact on patient outcomes. There were 3 distinct types of temporal segments for each patient. They considered observations up to the second week after discharge, treating each week as a distinct observation [42].

Feng et al [44] evaluated the feasibility of daily step count monitoring and the association between step counts and treatment-emergent symptoms in patients with prostate cancer. As shown in Table 1, the preprocessing technique could be summarized as follows: (1) a decline of 1000 steps or more as a binary predictor and (2) time window segmentation [44]. Jacobsen et al [48] impacted medical literature by proposing self-supervised contrastive learning methods for hematological malignancy treatments. Noninvasive monitoring of vital signs and physical activity was recorded within serious clinical complications in the input data set [48]. Data downsampling was the selected preprocessing technique to eliminate physical interruptions [48]. These studies collectively illustrated diverse data transform methods, such as feature selection, time segmentation, domain transformation, and time windowing, to enhance wearable device data quality, making them more suitable for AI/ML modeling aimed at predicting patient outcomes in cancer care. In addition, these findings have leveraged a range of wearable technologies and AI/ML methods to advance cancer care. Techniques, such as peak detection and Fourier transform have been used for data preprocessing, supporting applications that include postoperative rehabilitation, physical activity classification, prediction of treatment outcomes, and assessment of cancer-specific mortality. These studies highlight the potential of integrating high-dimensional wearable data with clinical information to enhance patient monitoring and prognosis.

### Data Normalization and Standardization

Barber et al [47] assessed the feasibility of postoperative intervention for patients with gynecologic cancer in a manner similar to Zhao et al [31], incorporating patient-reported outcomes and wearable activity data and also opting for standardization and normalization of preprocessing methods. Finally, Li et al [49] proposed a method using multimodel decision fusion based on multisource data for physical fitness assessment for patients with cancer. They enriched the raw data by using Baseline, Synthetic Minority Over-sampling Technique, random oversampling, adaptive synthetic oversampling, and Mahalanobis Distance and Boundary Constraints. The interval scaling method and *z* score standardization after segmentation are the common methods in the study by Li et al [49]. These additional investigations used tailored data preprocessing approaches to further refine the quality of wearable device data for subsequent analysis (eg, data partitioning for training and testing).

Moscato et al [32] proposed an automatic pain assessment for patients with cancer (21 in total) by using the Empatica wristband. Because all physiological signals were recorded at different sampling rates, different-order Butterworth filtering with different cutoff frequencies was the data enrichment selected method [32]. Each pulse was normalized with the *z* score procedure and processed with an automated algorithm that detects pulses suitable for heart rate variability analysis and derived metrics [32]. Liu et al [37] aimed to develop an unsupervised personalized sleep-wake identification algorithm using multistage data to explore the benefits of incorporating heart rate metrics and actigraphy data in these types of algorithms for the general population. After nonwear exclusion, there were 14 participants whose data qualified for analysis; 5 (36%) had high cholesterol, 6 (43%) participants had hypertension, 3 (21%) had cancer, 2 (14%) had diabetes mellitus, and 1 (7%) have had a stroke. They preprocessed the step count data, and 2 schematic ML-based models were designed by following the Markov model’s fundamentals. To facilitate the fusion of step count and heart rate data in the models, downscaling was used to deal with the multigranularity data [37]. In addition, imputation techniques were implemented. Tedesco et al [38] explored the prediction of cancer-specific mortality over a 2- to 7-year period using a data set from a longitudinal study of 2291 70-year-old Swedish patients, integrating wearable and electronic health record data. They applied standardization and normalization preprocessing techniques within imputation.

Vets et al [43] aimed to determine the accuracy of a pretrained laboratory-based MLM to distinguish functional from nonfunctional arm motions through home interventions of survivors from breast cancer populations. From the accelerometer data, functional activity was defined using two separate methods: (1) the counts threshold method, and (2) a pretrained MLM [43]. Activity counts were calculated from the raw acceleration data [43]. The outcome “total minutes active” was calculated as the sum of the 1-second epochs where the count threshold exceeded 1 [43]. Data normalization was the final step before fitting AI/ML models. van den Eijnden et al [45] created a model that predicted continuous recovery scores (regressors) in perioperative care in the hospital and at home for objective oncology-based decision-making. They preprocessed data by obtaining a balanced split in which they equally divided the demographic predictors and surgery type into 2 groups by splitting the patients 10,000 times [45]. Finally, authors standardized features by scaling the data to a normal distribution with a mean of 0 and a unit variance [45]. S et al [46] introduced a noninvasive wearable device developed as an adjunct to current modalities to assist in the detection of breast tissue abnormalities in any type of breast tissue. In the study, data normalization and outliers’ removal were the data transformation methods to enrich the quality of the collected temperature data.

### Data Cleaning

Liu et al [30] aimed to investigate the potential of using wearable devices and AI/ML to predict death events among patients with terminal cancer. To improve the model training, the authors used imputation techniques [30]. The data set was a combination of demographic, clinical, and wearable device data [30]. Davoudi et al [36] conducted a study comparing various accelerometer placements in classifying physical activity and associated energy expenditure among older adults. Of the 93 participants who completed the study, 27 (29%) were identified with a range of cancer diagnoses. Raw data were cleaned using bias reduction and eventually transformed by activity location and vector magnitude calculation [36]. Similarly, Patel et al [40] sought to enhance prognostic tools by combining ML analysis of actigraphy, sleep data, and routine clinical data with a missing data imputation technique within averaging. Asghari [41] proposed an internet of things–based predicting model to predict colorectal cancer in older adults. The data preprocessing phase was required to clean the sensed medical internet of things data from the inconsistencies and the noises for the data mining phase [41]. Outliers’ removal was the initial step selected for preprocessing.

**Figure 4:**
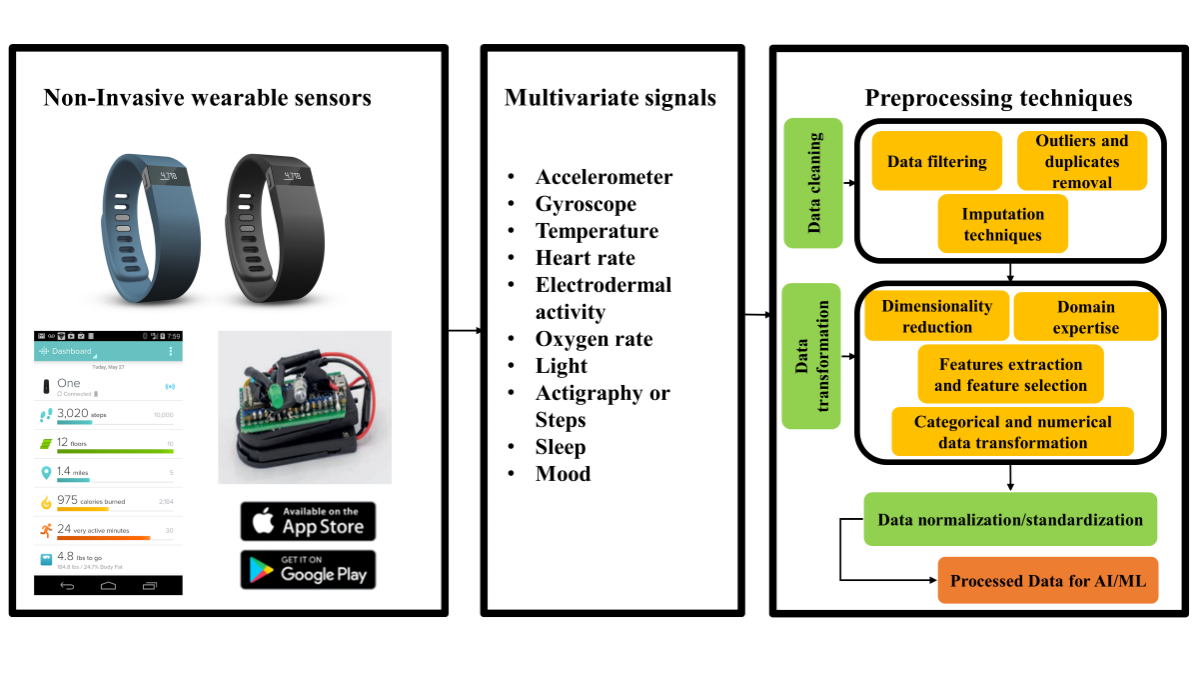
A general framework for data preprocessing techniques used to make noninvasive data collected from mobile health and wearable sensor artificial intelligence and machine learning (AI/ML) ready in cancer monitoring applications.

Accordingly, we proposed a generalized preprocessing framework that comprises all 3 major data preprocessing themes (Figure 4), reflecting the core elements that were consistently reported across studies.

## Discussion

### Principal Findings

In this paper, we conducted a scoping review of the preprocessing techniques applied to wearable sensor data in cancer care. Our findings revealed a significant rise in the use of wearable sensors for patient monitoring, along with an increase in preprocessing methods for data analysis over the past 5 years. This likely stemmed from recent advancements in sensor technology, greater emphasis on personalized and remote patient care, the rising prevalence of big data analytics in health care, and increasing recognition of real-time health data for precision oncology.

Data transformation emerged as the most reported preprocessing technique, representing approximately 60% (12/20) of the literature findings. Most studies relied on data from commercially available products, except a study by Zhao et al [31], which assessed a prototype’s efficiency in a small cohort. While published studies describing preprocessing methods for wearable devices are growing, the diagnoses being studied remain sparse and generally limited to single disease types or settings.

The physiological data captured from wearables are typically noisy, contain missing values, have outliers, redundant features, and erroneous measurements [50,51]. On the basis of the literature review in this paper, we found that various data cleaning procedures are used to clean the wearable sensor data, including data smoothing techniques (ie, moving average and exponential moving average) to reduce short-term signal artifacts and remove noise, removing duplicate entries, detection and removal of erroneous measurements due to sensor malfunctioning or losing contact of the sensor with skin or wearing the watch on incorrect body location, and outlier removal. The outlier removal for wearable data [52] in the reviewed studies consists of the range inspection of physiological parameter values with the clinically relevant range or developing a threshold using statistical techniques to detect outliers. Finally, missing data imputation is a critical component of data cleaning due to their ability to handle complex missing patterns as demonstrated in wearable-based data [53-57].

Our review suggests that the data cleaning procedures should be carefully inspected and applied based on the data captured from the wearables, as the captured data will produce false conclusions and predictions without proper data cleaning procedures, which is not acceptable in clinical research. In addition, the outliers’ removal should be based on data behavior and domain knowledge, as a region of anomaly is often within the boundaries of normal patterns of physiological data; for example, for the heart rate data, the normal behavior might evolve, which can be considered anomalous behavior, and the removal of data points leads to the loss of critical data. A generalized, automated, and adaptive data cleaning procedure is required for the wearable data to address the issues that arise due to improper data cleaning.

Time-series segmentation is the most used data transformation technique in wearable research identified in the review, necessitated by the multivariate nature of the data and varying sampling rates. Segmentation can be based on study outcomes, such as daily, hourly, or minute-by-minute intervals. Our review indicates that the optimal time window size for segmentation must be determined through experimentation to achieve the best performance results. This window size varies across different cancer cohorts and should be tailored to the specific data set rather than relying solely on literature. The granularity of time segmentation also affects feature extraction. For instance, summary statistics like mean, median, SD, and minimum, and maximum differ when calculated for daily versus hourly or minute-by-minute windows. The reviewed literature [58-60] also explores additional feature types, including frequency domain features and linear and nonlinear features.

Data compliance is another major challenge in wearable studies and has a profound impact on the study outcomes. Physiological data captured from wearables are highly variable [61] and have high noncompliance rates by the participants. The participants’ compliance determines the validity of the data collected from the wearables and their utility. Different thresholds are established for various parameters, such as daily wear time or step counts to filter or preprocess the data [62-64]. This scoping review suggests that we should strive to develop algorithms for standardizing the physiological metrics collected, which includes establishing thresholds for data inclusion based on compliance, filtering data based on adequate wearable wear time in study participants undergoing cancer per day and per week, percentage of days on which wearable was worn by the participants, inclusion and exclusion of data due to participant wearable synchronization issues, etc. ML techniques can be exploited to automate the data compliance assessments for different data extracted in different types of cancer.

Finally, data normalization is critical to developing AI/ML-ready data for the wearable studies. The data scaling helps not only in building efficient and accurate MLMs but also removes the effect of different scales and ranges in the model prediction. Our review suggests that researchers should identify the appropriate normalization technique for their study and understand the data distribution and model results before and after applying these techniques.

In summary, this scoping review identified 3 main categories of preprocessing techniques: data transformation, data normalization and standardization, and data cleaning, that have been applied to wearable sensor data in cancer care. While these techniques are commonly used to prepare data for AI/ML analysis, there is a lack of standardization in their implementation and limited evidence of their comparative effectiveness. Moreover, wearable sensor data are highly unstructured, complex, and messy because it is generated continuously and with high frequency (thousands of observations per second), leading to rich streams of time-series data. Thus, there is an urgent need to develop novel preprocessing procedures and frameworks, enhancing data quality and data readiness for AI/ML applications in cancer research. Future work should focus on developing validated preprocessing pipelines and benchmarking their impact on AI/ML model performance across diverse cancer populations and wearable devices. By providing a generalizable framework, we aim to accelerate the development of AI/ML models in not only cancer care but also potentially other areas of health care that leverage wearable sensor data. Researchers and clinicians can adapt this framework to their specific needs, promoting standardization while allowing for necessary customization.

### Preprocessing Techniques for General mHealth Applications

Preprocessing techniques have been a considerable topic of interest in the research community within its integration with the mHealth concept [65-67]. For example, cardiovascular diseases and diabetes are 2 conditions that have benefited from mHealth tools. In a study by Qaisar et al [68], an efficient method for the diagnosis of arrhythmia based on electrocardiogram inputs was proposed. The method combined multivariate processing, wavelet decomposition, frequency content-based subband coefficient selection, and ML techniques for preprocessing. In a study by Efat et al [69], a smart health monitoring tool for patients with diabetes was introduced. The objective of the authors was to use continuous sensor monitoring and processing with neural networks to provide a continuous evaluation of the patient’s health risk status by considering the patients’ noninvasive biometric data [69]. To improve data quality, the authors used data transformation. Photoplethysmography has been used for blood pressure monitoring by incorporating the mHealth concept [70]. The authors collected photoplethysmography signal data from smartphones and passed them through a high-pass filter with a cutoff frequency of 0.5 Hz. To filter out unwanted peaks and create a smooth signal, a moving average filter with a span of 5 data points was applied to the signals before peak detection was performed [70]. Peak detections were implemented by finding the local maximum values in the signals [70]. The incorporation of mHealth technology has brought several efficient alternatives for health care engineering. In addition, it becomes a challenging factor while addressing data quality issues. The general health care sector has experienced irregularities in converting raw data to suitable formats, there is not an exceptional case in cancer monitoring.

### Proposed Preprocessing Framework

To address the challenges and limitations identified in the reviewed literature, we propose a general preprocessing framework to develop AI/ML-ready data for mHealth cancer monitoring applications. Figure 4 summarizes this framework for noninvasive physiological monitoring data analysis. While our framework is conceptually applied within the setting of general oncology monitoring to fit AI/ML models, it could also be applied in other disease settings by following the key elements and steps of data preprocessing techniques.

Our proposed framework (Figure 4) synthesizes the best practices identified in this review, offering a standardized approach to preprocessing wearable sensor data. The framework’s strength lies in its flexibility and broad applicability. While the framework was developed based on cancer care applications, its fundamental components, data cleaning, data transformation, and data normalization and standardization, are relevant to a wide range of chronic diseases that can benefit from continuous monitoring via wearable sensors. By extracting raw wearable-based data from a real-world scenario, as shown in this paper using the cancer care setting, researchers should be able to reproduce available preprocessing solutions to other settings that leverage wearable sensor data. For instance, the data cleaning techniques identified in cancer studies, such as handling missing data and removing artifacts, are equally crucial in preprocessing data for heart disease or diabetes monitoring. Similarly, the data transformation methods, including feature extraction and dimensionality reduction, can be adapted to extract relevant biomarkers for various conditions. The framework’s emphasis on data normalization and standardization ensures that regardless of the specific disease context, the preprocessed data will be suitable for AI/ML applications.

Data captured from wearable sensors (eg, sleep parameters, heart rate, and steps) are unique in that they are collected passively, nonobtrusively, and continuously in real-world settings [71]. For cancer applications, the identification of noninvasive biomarkers is an attractive tool for possibly predicting clinical outcomes [72]. However, current challenges of applying AI/ML techniques in the cancer research setting include data quality issues, data dimensionality, diverse data types, dynamic evolution of disease states, lack of labeled data, frequent and irregular data sparsity, and data integration issues [73]. Noninvasive wearables, such as fitness trackers, smartwatches, and many medical monitoring devices, are built using standardized design and manufacturing processes. These standard processes pertain to aspects like how data are sampled (sampling rate), how the wearables are constructed (structural aspects), and how complex the devices are. Because of these standardized methods, wearable devices can operate in a manner that captures and provides data frequently, often in real time. This continuous stream of data means that wearables are consistently generating much information. Wearable technologies are still in their infancy in cancer research because they have not been widely implemented on patients diagnosed with oncology diseases. In addition, they still face challenges in being effectively used for cancer research because of difficulties in data collection, limited types of data captured, and the scattered nature of the data storage.

### Strengths and Limitations of the Review and Preprocessing Techniques

Our review provides a valuable synthesis of current preprocessing practices for wearable sensor data in cancer applications and highlights key opportunities for standardization and future research. By transparently reporting our methods and potential biases, we aim to support the interpretability and trustworthiness of our findings. Prior research has primarily focused on ML methods rather than emphasizing on standardized preprocessing techniques to make the data AI/ML ready. Key strengths and limitations are summarized in Multimedia Appendix 3. In addition, we point out potential factors that may influence the validity of our scoping review.

First, despite our comprehensive search strategy across multiple databases, it is possible that some relevant studies were not captured, particularly if they were published in nonindexed journals or as gray literature. However, we believe the risk of missing significant preprocessing methodologies is low given the breadth of our search and focus on peer-reviewed articles.

Second, categorizing preprocessing techniques required some subjective interpretation, as nomenclature was not always consistent across studies. We mitigated this by having multiple authors independently classify techniques and resolve discrepancies through discussion. Nonetheless, some overlap between categories may remain. The framework we proposed offers a generalizable taxonomy but should be further validated and refined as the field evolves.

Third, our analysis was limited to assessing the reported preprocessing workflows in each study. Without access to the underlying data sets and code, we could not directly compare the effectiveness or reproducibility of different techniques. Quantitative benchmarking of preprocessing methods on standardized wearable data sets would be a valuable direction for future work to provide more objective guidance for researchers.

### Conclusions

Herein, we conducted a scoping review of preprocessing techniques by focusing exclusively on enhancing raw data from wearables before fitting AI/ML models. Recently, there has been a worldwide interest in the data quality improvement elements in the biomedical area. Our review identified 3 different preprocessing categories applicable to cancer care. Data preprocessing plays a fundamental role in the knowledge discovery from analyzing cancer-related data, especially when data are captured from wearables. A general framework within conventional preprocessing tasks, including data cleaning, data transformation, and data normalization and standardization, has been proposed with a detailed preprocessing pipeline well described. However, due to the diversity of oncology diseases, we validated the availability of significant challenges in preprocessing technique implementation for AI/ML readiness. These methods can bring significant research outcomes across the enhancement of wearable data while addressing data quality issues through different data sets with diverse specifications. The general preprocessing framework proposed in this study represents a significant step toward standardizing the preparation of wearable sensor data for AI/ML applications. While developed in the context of cancer care, its principles are broadly applicable and adaptable to other chronic diseases requiring continuous monitoring. Future research should focus on validating and refining this framework across diverse health care contexts, potentially leading to more efficient and effective use of wearable sensor data in precision medicine.

## Appendix

Multimedia Appendix 1 Search queries.

Multimedia Appendix 2 PRISMA-ScR (Preferred Reporting Items for Systematic reviews and Meta-Analyses extension for Scoping Reviews) checklist.

Multimedia Appendix 3 Strengths and Limitations of Preprocessing Approaches.

## Abbreviations

AI/ML: artificial intelligence and machine learning
mHealth: mobile health
MLM: machine learning model
PRISMA: Preferred Reporting Items for Systematic Reviews and Meta-Analyses

## Data Availability

All data generated or analyzed during this study are included in this published article and its supplementary information files.
