# Supplementary material for: Data Preprocessing Techniques for AI and Machine Learning Readiness: Scoping Review of Wearable Sensor Data in Cancer Care
Source: JMIR Mhealth Uhealth. 2024 Sep 27;12:e59587. doi: 10.2196/59587 (PMC11470224; doi:10.2196/59587)
Supplement: Multimedia Appendix 1 [file mhealth_v12i1e59587_app1.docx]

**Supplemental Table 1.** Final Search Queries for each database.

| **#** | **Search Query in PubMed Database** | **Filters Applied** | **Results** |
| --- | --- | --- | --- |
| 1 | ("preprocessing"[tw] OR "pre-processing"[tw] OR "preparation"[tw] OR "processing"[tw] OR "signal processing"[tw] OR "analytics"[tw]) AND ("AI"[tw] OR "Artificial Intelligence"[tw] OR "Deep learning"[tw] OR "machine learning" [tw] OR "AI/ML"[tw]) AND ("mHealth"[tw] OR "mobile health*"[tw] OR "mobile app*"[tw] OR "mobile application*"[tw] OR "health app*"[tw]) | Last 5 years, Free Full Texts | 248 |

| **#** | **Search Query in Embase Database** | **Filters Applied** | **Results** |
| --- | --- | --- | --- |
| 1 | ('mobile application'/exp OR 'wearable computer'/exp OR 'apple watch*':ab,ti OR 'digital health':ab,ti OR 'e health':ab,ti OR 'mobile health':ab,ti OR 'smart phone*':ab,ti OR 'smart ring*':ab,ti OR 'smart watche*':ab,ti OR wearable*:ab,ti) AND ('artificial intelligence'/exp OR 'ai app':ab,ti OR 'ai application':ab,ti OR 'ai apps':ab,ti OR 'ai health care':ab,ti OR 'ai healthcare':ab,ti OR 'alphago':ab,ti OR 'artificial general intelligence':ab,ti OR 'artificial intelligence':ab,ti OR 'artificial narrow intelligence':ab,ti OR 'artificial neural network':ab,ti OR 'artificial social intelligence':ab,ti OR 'augmented intelligence':ab,ti OR 'chatbot':ab,ti OR 'chatgpt':ab,ti OR 'computer neural networks':ab,ti OR 'conversational ai':ab,ti OR 'deep learning':ab,ti OR 'deep mesh':ab,ti OR 'deep neural network':ab,ti OR 'dental ai':ab,ti OR 'expert systems':ab,ti OR 'fuzzy logic':ab,ti OR 'generative ai':ab,ti OR 'generative pre-trained transformer':ab,ti OR 'generative pretrained transformer':ab,ti OR 'google bard':ab,ti OR 'gpt-3.5':ab,ti OR 'gpt-3':ab,ti OR 'gpt-4':ab,ti OR 'gpt':ab,ti OR 'heuristic':ab,ti OR 'intelligence augment':ab,ti OR 'knowledge base':ab,ti OR 'knowledge engineer':ab,ti OR 'large language model':ab,ti OR 'logistic model':ab,ti OR 'logistic models':ab,ti OR 'logistic regression':ab,ti OR 'machine intelligence':ab,ti OR 'machine learning':ab,ti OR 'medical ai':ab,ti OR 'naive bayes':ab,ti OR 'natural language processing':ab,ti OR 'natural language understanding':ab,ti OR 'open ai':ab,ti OR 'optical character recognition':ab,ti OR 'random forest':ab,ti OR 'recurrent neural network':ab,ti OR 'robotic process automation':ab,ti OR 'sentiment analys*':ab,ti OR 'support vector':ab,ti OR llm:ab,ti OR nlp:ab,ti OR openai:ab,ti OR 'pre processing':ab,ti OR preprocessing:ab,ti) AND ('neoplasm'/exp OR 'tumor diagnosis'/exp OR 'bone marrow transplant*':ab,ti OR cancer*:ab,ti OR carcinoma*:ab,ti OR leukemia*:ab,ti OR lymphoma*:ab,ti OR malignan*:ab,ti OR myeloma*:ab,ti OR neoplasm*:ab,ti OR neoplastic:ab,ti OR oncolog*:ab,ti OR tumor*:ab,ti OR tumour*:ab,ti) | Last 5 years | 475 |

| **#** | **Search Query in Scopus Database** | **Filters Applied** | **Results** |
| --- | --- | --- | --- |
| 1 | TITLE-ABS-KEY(( "apple watches" OR "digital health" OR "e health" OR "mobile app" OR "mobile application" OR "mobile health" OR "smart phone" OR "smart phones" OR "smart ring" OR "smart rings" OR "smart watch" OR "smart watches" OR fitbit OR fitbits OR smartphone OR smartphones OR smartring OR smartrings OR smartwatch OR smartwatches OR wearable OR wearables ) AND ( "ai app" OR "ai application" OR "ai apps" OR "ai health care" OR "ai healthcare" OR "artificial general intelligence" OR "artificial intelligence" OR "artificial narrow intelligence" OR "artificial neural network" OR "artificial social intelligence" OR "augmented intelligence" OR "chatbot" OR "chatgpt" OR "computer neural networks" OR "conversational ai" OR "deep learning" OR "deep mesh" OR "deep neural network" OR "expert systems" OR "fuzzy logic" OR "generative ai" OR "generative pre-trained transformer" OR "generative pretrained transformer" OR "google bard" OR "gpt-3.5" OR "gpt-3" OR "gpt-4" OR "gpt" OR "heuristic" OR "intelligence augment" OR "knowledge base" OR "knowledge engineer" OR "large language model" OR "logistic model" OR "logistic models" OR "logistic regression" OR "machine intelligence" OR "machine learning" OR "medical ai" OR "naive bayes" OR "natural language processing" OR "natural language understanding" OR "open ai" OR "optical character recognition" OR "random forest" OR "recurrent neural network" OR "robotic process automation" OR "sentiment analys*" OR "support vector" OR llm OR nlp OR openai OR pre-processing OR preprocessing ) AND ( "bone marrow transplant" OR cancer OR cancers OR carcinoma OR leukemia OR lymphoma OR malignancy OR myeloma OR neoplasm OR neoplasms OR neoplastic OR oncology OR tumor OR tumor )) | Last 5 years, English, Full Articles only | 428 |

| **#** | **Search Query in IEEE Xplore Database** | **Filters Applied** | **Results** |
| --- | --- | --- | --- |
| 1 | (smartwatches OR wearable devices OR biomedical OR physiological) AND (cancer detection OR tumor OR cancer OR oncology) AND (signal processing OR biomedical signal processing OR processing) AND (artificial intelligence OR machine learning) | Last 5 years, Conferences, and Journals | 996 |
