# Supplementary material for: Data Preprocessing Techniques for AI and Machine Learning Readiness: Scoping Review of Wearable Sensor Data in Cancer Care
Source: JMIR Mhealth Uhealth. 2024 Sep 27;12:e59587. doi: 10.2196/59587 (PMC11470224; doi:10.2196/59587)
Supplement: Multimedia Appendix 3 [file mhealth_v12i1e59587_app3.docx]

**Table S3.** Strengths and Limitations of Preprocessing Approaches.

| Limitations | Strengths | Preprocessing Techniques |
| --- | --- | --- |
| - Requires domain expertise to design appropriate transformations.  - May discard potentially useful information in the raw signals. | - Converts unstructured high-dimensional sensor data into a more compact and informative format tailored for the modeling task.  - Can extract relevant features and reduce dimensionality. | Data Transformation |
| - Does not address other data quality issues like noise and missingness.  - Inappropriate scaling can reduce signal variance. | - Creates a more uniform feature space.  - Improves convergence of machine learning algorithms.  - Enhances comparability between features on different scales. | Data Normalization and Standardization |
| - Requires careful application to avoid introducing bias by removing important natural variability.  - Over-cleaning risks yielding data that is unrepresentative of the true physiological state. | - Improves the reliability of datasets used to train AI/ML(a) models.  -Reduces the impact of erroneous values such as missing data, outliers, and inconsistencies. | Data Cleaning |
| - Increased complexity in preprocessing pipeline design.  - Potential for compounding limitations if not applied judiciously. | - Can be complementary, addressing multiple aspects of data quality.  - Allows researchers to tailor preprocessing to the specific characteristics of their wearable data and modeling objectives. | Combined Approaches |

(a): AI/MI: artificial intelligence and machine learning.
